# Modeling of Photodynamic Self-Oscillation Based on a Suspended Liquid Crystal Elastomer Ball System

**DOI:** 10.3390/polym16223119

**Published:** 2024-11-07

**Authors:** Leilei Li, Yuntong Dai, Jun Zhao

**Affiliations:** School of Civil Engineering, Anhui Jianzhu University, Hefei 230601, China; lileilei@stu.ahjzu.edu.cn (L.L.); daiytmechanics@ahjzu.edu.cn (Y.D.)

**Keywords:** liquid crystal elastomer, self-oscillation, photodynamic, ball

## Abstract

Self-oscillation enables continuous motion by transforming constant external stimuli into mechanical work, eliminating the necessity for supplementary control systems. This holds considerable promise in domains like actuators, wearable devices and biomedicine. In the current study, a novel suspended liquid crystal elastomer (LCEs) ball system consisting of a light-responsive hollow LCE ball and an air blower is constructed. Stable illumination allows for its continuous periodic oscillation. Drawing from the theoretical model in conjunction with the dynamic LCE model, the control equations for the system are established, and its dynamic motion characteristics are explored from theoretical viewpoint. The numerical calculations suggest that two motion patterns are present, i.e., hovering and self-oscillatory patterns. The critical conditions required to initiate the transition between two motion patterns are quantified for different system parameters. As evidenced by the outcomes, manipulating the light intensity, damping coefficient, contraction coefficient, air density, gravitational acceleration, bottom illumination zone height, characteristic coefficient and vertical wind speed at the blower outlet facilitates precise control over the motion patterns as well as the amplitude and frequency. With its simple structure, customizable dimensions, remote activation and active manipulation, this system may potentially change the design approach for energy harvesting, microsensors and aerial vehicles.

## 1. Introduction

The occurrence of self-oscillation is prevalent in both routine life and engineering practices [1,2]. Through the consistent uptake of energy from external stimuli, it can maintain a continuous periodic motion pattern without the necessity for complex controllers or self-supplied power supply [3,4,5], thus reducing energy consumption and system complexity. Moreover, self-oscillation typically exhibits strong robustness, with its frequency typically being related to its own attributes [6] and not being reliant on the initial conditions. The superiority of self-oscillation has led to its extensive adoption in diverse fields, including mechanical logic devices, energy harvesting, signal sensors, soft robotics, and bionic design [7,8,9,10,11,12,13,14,15,16,17,18].

In recent years, more and more active materials with unique and enhanced properties have been discovered, such as hydrogels [19,20], liquid crystal elastomers (LCEs) [21,22,23], ionic gels [24,25], dielectric elastomers [26] and thermally responsive polymer materials [27]. These materials exhibit varied responses to different stimuli, i.e., light [28], electric fields [29], magnetic fields [30] and temperature [31]. By employing the properties of these active materials, a number of self-oscillatory systems and various self-sustained motion patterns have been established, e.g., oscillating [32,33,34], vibration [35,36], swinging [37,38], rotating [39,40], contracting [41,42], buckling [43,44,45], bending [46,47,48,49,50], jumping [51,52,53,54,55], floating [56], rolling [57] and spinning [58,59,60,61]. Concurrently, these self-oscillatory motions rely on a range of established feedback mechanisms, which include the coupling of a chemical reaction with large deformations [62], photothermal surface tension gradient [63] and self-shadowing [64,65].

LCEs exhibit responsiveness to a diverse array of stimuli, among which light stands out for its persistence, high precision and easy control without physical contact [42,66]. The synthesis of light-responsive LCEs incorporates rod-like liquid crystal molecules with anisotropic properties and stretchable long-chain polymers [67]. Upon receiving stimulation from an external field, the liquid crystal monomer molecules engage in rotational or phase transitions, which modify the conformation, resulting in noticeable deformations. Accordingly, light-responsive LCEs have the benefits of rapid deformation response, large inherent deformation and shape memory effect [68]. The deformation characteristics of LCEs have inspired the theoretical construction of multiple self-sustained motion systems, like the self-excited swing of a three-dimensional pendulum and a semi-rotary motor [36,37,38,39,40,41,42,43,44,45,46,47,48,49,50,51,52,53,54,55,56,57,58,59,60,61,62,63,64,65,66,67,68,69]. These self-oscillatory systems fully exploit the properties of LCEs, i.e., contraction in the presence of light and deformation recovery in the absence of light. On this basis, the utilization of light-responsive LCEs in photodynamic self-oscillation offers various promising opportunities in the field of actuators [70,71].

Despite previous research on self-oscillatory systems, their overall structure remains intricate and difficult to manage, posing challenges for practical uses. Hence, it is imperative to develop additional motion patterns to satisfy diverse potential applications and effectively handle complex scenarios to meet the growing demand [72,73]. Inspired by contraction–expansion-induced self-oscillations, the Bernoulli principle and the Coanda effect, and by observing the example of a table tennis ball hovering in the air under the strong airflow from an air blower, we established a novel oscillator composed of a hollow LCE ball and an air blower to study its periodic self-sustaining property under stable illumination. The existing systems specifically study and analyze a unique class of self-sustaining chaotic jump systems, revealing their complex chaotic mechanisms and behaviors [53]. In this paper, we mainly analyze the self-sustained oscillations in the air by adjusting the critical parameter that allows the LCE ball to go from hovering to self-oscillatory patterns and by changing the wind speed of the airflow, thus affecting the deformation and movement trajectory of the LCE ball, showing the interaction of the fluid dynamics and the liquid crystal elastomer. With its simple structure, customizable dimensions, remote activation and active manipulation, this system may potentially change the design approach for energy harvesting, microsensors and aerial vehicles.

The following contents are included in this paper: Section 2 outlines the theoretical model and governing equations for the suspended LCE ball system utilizing the dynamic LCE model. Section 3 provides an extensive discussion on the two distinct motion patterns that characterize the system, i.e., the hovering and self-oscillatory patterns, as well as the mechanism that facilitates self-oscillation. In Section 4, we present the visual representations and thoroughly assess the impact of diverse parameters on the LCE ball system. Finally, Section 5 offers a concise overview of this paper.

## 2. Theoretical Model and Formulation

In this section, we first introduce a theoretical model of a suspended LCE ball system consisting of a hollow LCE ball and an air blower under stable illumination. Then, we clarify the governing equations relevant to the system, the number fraction of cis-isomers within the LCE and the dimensionless treatment of system parameters and governing equations and provide the approaches to solve them.

### 2.1. Self-Oscillatory Dynamics of LCE Ball System

**Figure 1:**
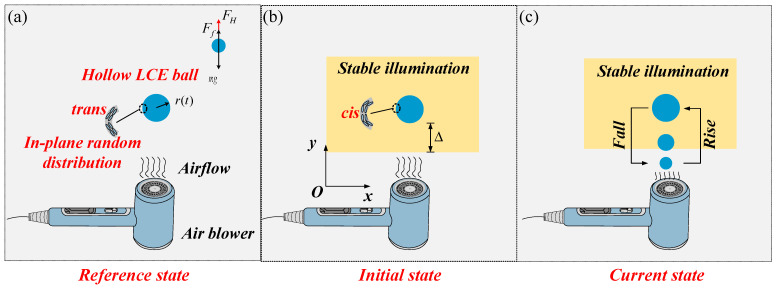
A schematic diagram of an LCE ball system containing a hollow LCE ball, an air blower and a light source: (**a**) the reference state, (**b**) initial state, and (**c**) current state. Under steady illumination conditions, the hollow LCE ball vibrates in a continuous periodic manner.

By observing the example of a table tennis ball hovering in the air under the strong airflow from an air blower, we established a novel oscillator composed of a hollow LCE ball and an air blower, which is capable of continuous oscillation under stable illumination. Figure 1 describes the photodynamic self-oscillations of the LCE ball system with a radius of $r\left(t\right)$. The model consists of a hollow LCE ball with mass m, an air blower and a light source. Given the Bernoulli principle and the Coanda effect, the hollow LCE ball is blown up and suspended in the air by the strong airflow generated by the air blower directly below it, as depicted in Figure 1a. In the initial state, the distance between the bottom surface of the hollow LCE ball and the lower edge of the illuminated zone is Δ, identified as the bottom illumination zone height, and a coordinate system is established, as plotted in Figure 1b. The LCE ball has photosensitive molecules like azobenzene molecules and is distributed randomly along the surface of the ball. When the hollow LCE ball is in the illuminated zone, the azobenzene liquid crystal molecule will change from a straight trans state to a bent cis state, and it undergoes light-driven contraction and becomes smaller in radius, thus falling vertically downwards. When the hollow LCE ball vibrates into the dark zone, the azobenzene liquid crystal molecule transitions from a bent cis state to a straight trans state. Subsequently, the recovery of the light-driven contraction strain leads to an expansion in its radius, which, in turn, propels it upward. Light energy absorption allows the LCE ball to counteract the damping dissipation, thereby resulting in continuous up-and-down oscillation, as shown in Figure 1c.

Given the force analysis in Figure 1a, the hollow LCE ball is subjected to buoyancy $F_{H}$, damping force $F_{f}$ and gravity mg. According to the Bernoulli principle and the Koanda effect, it can not only blow vertically, but also sideways. When blowing sideways, vertical and horizontal components will be generated, resulting in horizontal displacement, which causes the ball to vibrate obliquely upward. For the sake of simplifying the calculations, this system does not consider the effect of the measured air flow. Only air damping is considered. Therefore, the governing equation along the y-axis can be derived as follows:

(1) $$m\ddot{y}=-mg+F_{f}+F_{H},$$

where $\ddot{y}$ is the acceleration of the hollow LCE ball and g is the gravitational acceleration.

The damping force $F_{f}$ is calculated as follows:

(2) $$F_{f}=\beta \dot{y},$$

where $\dot{y}$ is the velocity of the hollow LCE ball.

The buoyancy $F_{H}$ from the airflow is calculated as follows:

(3) $$F_{H}=\frac{1}{2}p_{0}v_{l}^{2}\pi r^{2},$$

where $p_{0}$ is the air density, and $v_{l}$ is the air velocity of the hollow LCE ball.

(4) $$v_{l}=\frac{v_{0}}{1+{\left(\frac{y}{k}\right)}^{2}},$$

where $v_{0}$ is the vertical wind speed at the blower outlet, y is the relative position between the hollow LCE ball and the blower outlet and k is the characteristic coefficient of the air blower.

Substituting Equations (2)–(4) into Equation (1) yields the following:

(5) $$m\ddot{y}=-mg+\beta \dot{y}+\frac{1}{2}p_{0}{\left(\frac{v_{0}}{1+{\left(\frac{y}{k}\right)}^{2}}\right)}^{2}\pi r^{2}.$$

For the hollow LCE ball within the illumination, the formula for its radius is

(6) $$r(t)=r_{0}(1+\varepsilon_{r}(t)),$$

where $r_{0}$ is the initial radius of the hollow LCE ball and $\varepsilon_{r}(t)$ is the light-driven contraction strain of the LCE material.

Thus, Equation (5) can be rewritten as

(7) $$m\ddot{y}=-mg+\beta \dot{y}+\frac{1}{2}p_{0}{\left(\frac{v_{0}}{1+{\left(\frac{y}{k}\right)}^{2}}\right)}^{2}\pi {\left[r_{0}\left(1+\varepsilon_{r}\left(t\right)\right)\right]}^{2}.$$

It is reasonable to suggest that the number fraction $\phi \left(t\right)$ of cis-isomers in the LCE material is linearly correlated with the light-driven contraction strain $\varepsilon \left(t\right)$, which can be formulated as

(8) $$\varepsilon_{r}(t)=-C_{0}\phi (t),$$

where $C_{0}$ is the contraction coefficient.

### 2.2. The Evolution of the Cis Number Fraction

The light-driven contraction strain of the hollow LCE ball and its radius are determined by the cis number fraction $\phi \left(t\right)$ within the LCE. Yu et al. found that the LCE can undergo trans-to-cis isomerization when exposed to UV or laser light with a wavelength below 400 nm [74]. The cis number fraction depends on thermal excitation from the trans state to the cis state, thermally driven relaxation from the cis state to the trans state and light-induced trans-to-cis isomerization [75]. Frequently, the thermal excitation of the trans state to the cis state tends to be relatively small, making its effect inconsequential at room temperature. The following governing equation is employed to outline the evolution of the cis number fraction:

(9) $$\frac{\partial \phi }{\partial t}=\eta_{0}I_{0}(1-\phi )-\frac{\phi }{T_{0}},$$

where $I_{0}$ denotes the light intensity, $T_{0}$ is the thermal relaxation time from the cis state to the trans state and $\eta_{0}$ indicates the light absorption constant.

By solving Equation (9), the cis number fraction can be written as follows:

(10) $$\phi (t)=\frac{\eta_{0}{T_{0}}_{I}}{\eta_{0}T_{0}I+1}+\left(\phi_{0}-\frac{\eta_{0}{T_{0}}_{I}}{\eta_{0}T_{0}I+1}\right)\exp\left[-\frac{t}{T}(\eta_{0}T_{0}I+1)\right],$$

where $\phi_{0}$ refers to the initial cis number fraction.

This paper encompasses three distinct cases. In the first case, the hollow LCE ball is in the illuminated zone with $\phi_{0}=0$ initially; thus, Equation (10) can be modified as follows:

(11) $$\phi (t)=\frac{\eta_{0}T_{0}I}{\eta_{0}T_{0}I+1}\left\{1-\exp\left[-\frac{t_{1}}{T_{0}}(\eta_{0}T_{0}I+1)\right]\right\}$$

In the second case, i.e., the hollow LCE ball switching from the dark zone to the illuminated zone with transient $\phi_{0}=\phi_{dark}$, Equation (10) can be revised to

(12) $$\phi (t)=\frac{\eta_{0}T_{0}I}{\eta_{0}T_{0}I+1}+\left(\phi_{dark}-\frac{\eta_{0}T_{0}I}{\eta_{0}T_{0}I+1}\right)\exp\left[-\frac{t_{2}}{T_{0}}(\eta_{0}T_{0}I+1)\right].$$

In the third case, i.e., the hollow LCE ball switching from the illuminated zone to the dark zone (I=0) with transient $\phi_{0}=\phi_{light}$, Equation (10) can be rewritten as follows:

(13) $$\phi (t)=\phi_{light}\exp\left(-\frac{t_{3}}{T_{0}}\right).$$

where $t_{1}$, $t_{2}$ and $t_{3}$ are the durations of the current process, respectively, and $\phi_{dark}$ and $\phi_{light}$ are the *cis* number fractions at the switching moments from the dark zone to the illuminated zone and from the illuminated zone to the dark zone, respectively.

### 2.3. Nondimensionalization

The subsequent dimensionless quantities are outlined as follows: $\bar{\ddot{y}}=\frac{\ddot{y}T_{0}^{2}}{r_{0}}$, $\bar{\dot{y}}=\frac{\dot{y}T_{0}}{r_{0}}$, $\bar{y}=\frac{y}{r_{0}}$, $\bar{t}=\frac{t}{T_{0}}$, $\bar{g}=\frac{gT_{0}^{2}}{r_{0}}$, $\bar{\beta }=\frac{\beta T_{0}}{m}$, $\bar{T}=\frac{T}{T_{0}}$, $\bar{k}=\frac{k}{r_{0}}$, $\bar{\Delta }=\frac{\Delta }{r_{0}}$, $\bar{I}=I\eta_{0}T_{0}$, ${\bar{p}}_{0}=\frac{p_{0}r_{0}^{3}}{m}$, ${\bar{v}}_{0}=\frac{v_{0}T}{r_{0}}$ and ${\bar{F}}_{H}=\frac{F_{H}}{mg}$.

Accordingly, expressing Equation (7) in a dimensionless manner yields the following:

(14) $$\bar{\ddot{y}}=-\bar{g}+\bar{\beta }\bar{\dot{y}}+\frac{1}{2}{\bar{p}}_{0}{\left(\frac{{\bar{v}}_{0}}{1+{\left(\frac{\bar{y}}{\bar{k}}\right)}^{2}}\right)}^{2}\pi {\left[{\bar{r}}_{0}\left(1-C_{0}\phi \left(\bar{t}\right)\right)\right]}^{2},$$

where $\bar{\dot{y}}$ and $\bar{\ddot{y}}$ are the dimensionless velocity and acceleration of the hollow LCE ball, respectively.

The dimensionless treatment of the cis number fraction can be conducted in accordance with Equations (11)–(13). In the first case,

(15) $$\phi (\bar{t})=\frac{\bar{I}}{\bar{I}+1}\left\{1-\exp\left[-{\bar{t}}_{1}\left(1+\bar{I}\right)\right]\right\}.$$

In the second case,

(16) $$\phi (\bar{t})=\frac{\bar{I}}{\bar{I}+1}+\left(\phi_{dark}-\frac{\bar{I}}{\bar{I}+1}\right)\exp\left[-{\bar{t}}_{2}\left(\bar{I}+1\right)\right].$$

In the third case,

(17) $$\phi (\bar{t})=\phi_{light}\exp\left(-{\bar{t}}_{3}\right).$$

Meanwhile, Equation (2) can be converted into the subsequent dimensionless expression:

(18) $${\bar{F}}_{f}=\bar{\beta }\bar{\dot{y}}.$$

Equation (3) can be rewritten as

(19) $${\bar{F}}_{H}=\frac{1}{2}{\bar{p}}_{0}{\left(\frac{{\bar{v}}_{0}}{1+{\left(\frac{\bar{y}}{\bar{k}}\right)}^{2}}\right)}^{2}\pi {\left[{\bar{r}}_{0}\left(1-C_{0}\phi \left(\bar{t}\right)\right)\right]}^{2}.$$

In brief, Equations (14)–(17) describe the self-oscillation process of the LCE ball, among which the cis number fraction is coupled to the position of the hollow LCE ball. To derive the solutions for these differential equations with variable coefficients, we adopted the classical fourth-order Runge–Kutta method, and we first used the MATLAB (2016b) version for calculations, with the time step set at 0.001. Then, data processing and image generation were performed in the origin 2021 version and finally imported into the visio software (2016) for the in-processing of the image.

Based on the position $y_{i}$ of the hollow LCE ball and the *cis* number fraction $\phi_{i}$ at time $t_{i}$, we can calculate the buoyancy ${\bar{F}}_{Hi}$ and the damping force ${\bar{F}}_{fi}$ on the hollow LCE ball sequentially from Equations (18) and (19). Then, the position $y_{i+1}$ of the hollow LCE ball at time $t_{i+1}$ can be calculated using Equation (14). Time variations in the *cis* number fraction are obtained from Equations (15)–(17). Therefore, for the given parameters of $\bar{I}$, $C_{0}$, $\bar{\beta }$, $\bar{g}$, ${\bar{p}}_{0}$, $\bar{k}$, $\bar{\Delta }$ and ${\bar{v}}_{0}$, the dynamics of the system can be acquired through iterative calculations.

## 3. The Two Motion Patterns and Mechanism of Self-Oscillation

In this section, we describe two distinct motion mechanisms, identified as the hovering and self-oscillatory patterns. Subsequently, a thorough explanation on the underlying mechanism that facilitates self-oscillation is provided.

### 3.1. Two Motion Patterns

Before delving into the motion patterns, it is essential to first adjust the dimensionless parameters in the model and determine their typical values. With reference to prior experiments [76,77], Table 1 showcases the material properties along with the geometric parameters. Likewise, Table 2 includes the associated dimensionless parameters. These parameters will be employed to analyze the self-oscillation of the system under stable illumination.

**Figure 2:**
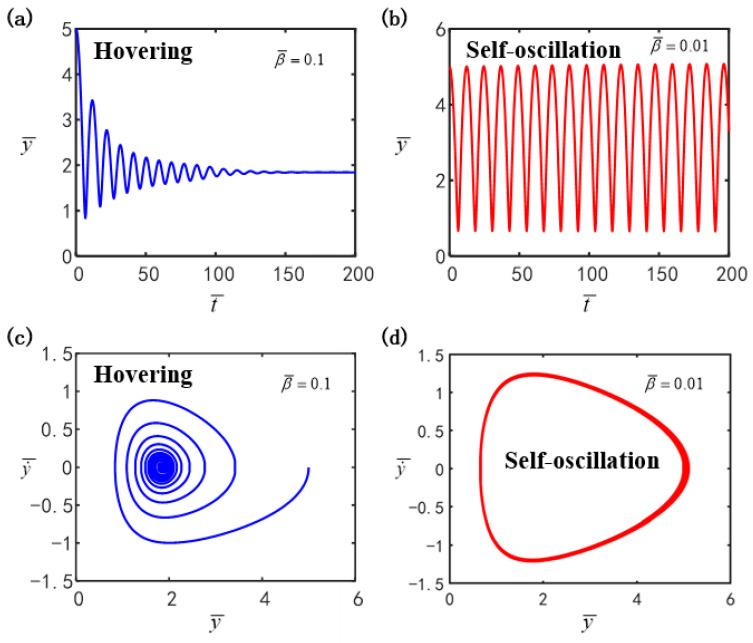
The time history and phase trajectory for the two motion patterns of the LCE ball system. (**a**,**c**) The hovering pattern ($\bar{\beta }=0.1$); (**b**,**d**) the self-oscillatory pattern ($\bar{\beta }=0.01$). The other parameters are $\bar{I}=0.4$, $\bar{g}=0.3$, $C_{0}=0.3$, ${\bar{p}}_{0}=0.5$, $\bar{\Delta }=3$, $\bar{k}=1$ and ${\bar{v}}_{0}=5$.

Figure 2 presents the two typical motion patterns of the LCE ball system under specific parameter combinations, i.e., the hovering pattern (Figure 2a,c) and the self-oscillatory pattern (Figure 2b,d). For the computation, the other parameters were determined as $\bar{I}=0.4$, $\bar{g}=0.3$, $C_{0}=0.3$, ${\bar{p}}_{0}=0.5$, $\bar{\Delta }=3$, $\bar{k}=1$ and ${\bar{v}}_{0}=5$. By numerically solving Equations (14)–(17), the time history curves and phase trajectory diagrams of the LCE ball system can be obtained. For $\bar{\beta }=0.1$, as plotted in Figure 2a,c, the hollow LCE ball vibrates for a period of time and then remains suspended due to the damping dissipation, which is named as the hovering pattern. For $\bar{\beta }=0.01$, as depicted in Figure 2b,d, the hollow LCE ball initially shifts from a static to an oscillatory pattern and gradually achieves a consistent oscillation amplitude as time progresses. Ultimately, the system demonstrates a continuous periodic self-oscillation, labeled as the self-oscillatory pattern. The hollow LCE ball counteracts the damping dissipation by harnessing illumination energy, thus allowing it to oscillate in a self-sustained manner.

### 3.2. Mechanism of Self-Oscillation

**Figure 3:**
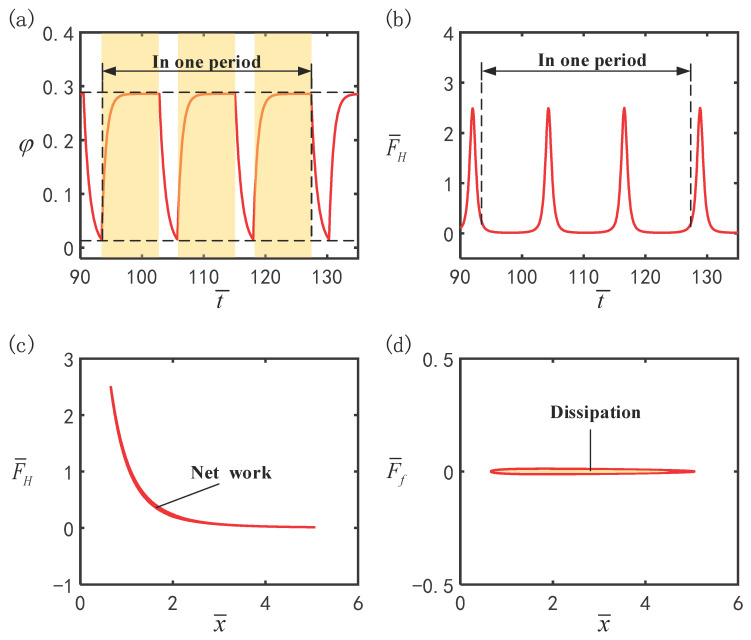
Mechanism of self-oscillation. (**a**) Time history of *cis* number fraction ϕ. (**b**) Time variation in buoyancy ${\bar{F}}_{H}$. (**c**) Displacement dependence of buoyancy ${\bar{F}}_{H}$. (**d**) Displacement dependence of damping force ${\bar{F}}_{f}$.

To understand the mechanism of self-oscillation and to clarify the way the system exploits the input light energy to counteract the damping dissipation, the dimensionless parameters were set to $\bar{I}=0.4$, $\bar{\beta }=0.01$, $\bar{g}=0.3$, $C_{0}=0.3$, ${\bar{p}}_{0}=0.5$, $\bar{\Delta }=3$, $\bar{k}=1$ and ${\bar{v}}_{0}=5$. Figure 3 plots the time history curves of four significant physical quantities for the representative case in Figure 2c,d. In Figure 3a, the cis number fraction ϕ experiences a gradual growth in the illuminated zone and a gradual decline in the dark zone, where the yellow region denotes the illuminated zone. The changes in buoyancy ${\bar{F}}_{H}$ that occur periodically with time for the hollow LCE ball are described in Figure 3b. Moreover, as depicted in Figure 3c, the correlation curve between buoyancy ${\bar{F}}_{H}$ and displacement forms a closed loop, and this enclosed region refers to the work performed by the buoyancy ${\bar{F}}_{H}$ on the hollow LCE ball in one period, quantified as 7.1 × 10^−2^. Likewise, the area surrounded by the curve in Figure 3d is the negative work performed by the damping force ${\bar{F}}_{f}$, with a magnitude of 7.1 × 10^−2^, which corresponds exactly to the net work performed by the buoyancy ${\bar{F}}_{H}$. Consequently, the work performed by the buoyancy precisely counteracts the negative work performed by the damping force of the system, thus enabling the hollow LCE ball to achieve a continuous and periodic self-oscillatory manner.

## 4. Effects of System Parameters on Self-Oscillation

According to the previous theoretical model and governing equations, eight system parameters are present, including $\bar{I}$, $C_{0}$, $\bar{\beta }$, $\bar{g}$, ${\bar{p}}_{0}$, $\bar{k}$, $\bar{\Delta }$ and ${\bar{v}}_{0}$. This section examines how these system parameters influence the frequency f and amplitude A of the self-oscillations.

### 4.1. Effect of Light Intensity

**Figure 4:**
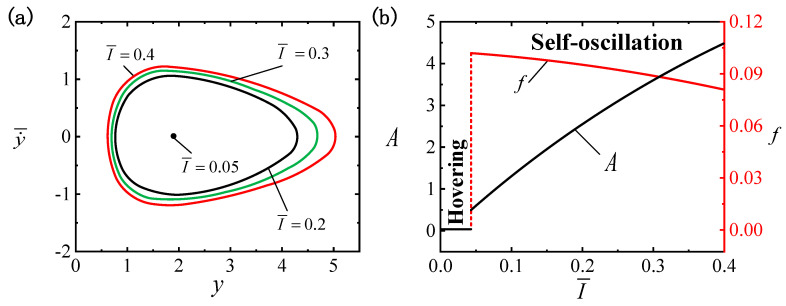
Effect of light intensity $\bar{I}$ on self-oscillation for $C_{0}=0.3$, $\bar{\beta }=0.01$, $\bar{g}=0.3$, ${\bar{p}}_{0}=0.5$, $\bar{\Delta }=3$, $\bar{k}=1$ and ${\bar{v}}_{0}=5$. (**a**) Limit cycles. (**b**) Effect of $\bar{I}$ on f and A. Enhanced $\bar{I}$ increases A and reduces f.

Figure 4 illustrates the effect of $\bar{I}$ on the LCE ball system, where the remaining parameters are $C_{0}=0.3$, $\bar{\beta }=0.01$, $\bar{g}=0.3$, ${\bar{p}}_{0}=0.5$, $\bar{\Delta }=3$, $\bar{k}=1$ and ${\bar{v}}_{0}=5$. Figure 4a depicts the limit cycles for different light intensities, among which the critical light intensity required to trigger the self-oscillatory pattern is identified as ${\bar{I}}_{0cr}\approx 0.05$. When $\bar{I}$ drops beneath the critical value, the input energy is inadequate to offset the damping dissipation, leading the system to persist in a hovering pattern. Conversely, the self-oscillatory pattern is triggered when ${\bar{I}}_{0}>0.05$, e.g., $\bar{I}=0.2$, $\bar{I}=0.3$ and $\bar{I}=0.4$. Figure 4b displays the influence of $\bar{I}$ on f and A. Enhanced light intensity brings an increased self-oscillation amplitude and reduced frequency, and it can be seen that adjusting $\bar{I}$ triggers different self-oscillating vibration effects. The conversion of absorbed light energy into mechanical energy leads to the occurrence of self-oscillation, and as $\bar{I}$ increases, more light energy is converted. Hence, the amplification of light intensity can be optimized for engineering applications of the self-oscillating suspended LCE ball system.

### 4.2. Effect of Contraction Coefficient

**Figure 5:**
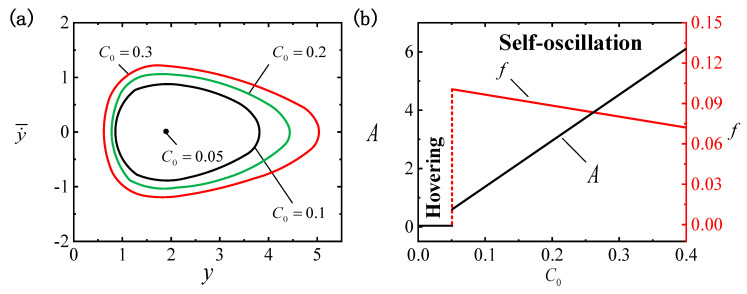
The effect of the contraction coefficient $C_{0}$ on self-oscillation for $\bar{I}=0.4$, $\bar{\beta }=0.01$, $\bar{g}=0.3$, ${\bar{p}}_{0}=0.5$, $\bar{\Delta }=3$, $\bar{k}=1$ and ${\bar{v}}_{0}=5$. (**a**) Limit cycles. (**b**) The effect of $C_{0}$ on f and A. When $C_{0}$ grows, A follows an ascending path, while f takes a descending path.

Figure 5 provides the effect of $C_{0}$ on the LCE ball system. The values for the other system parameters are set as follows: $\bar{I}=0.4$, $\bar{\beta }=0.01$, $\bar{g}=0.3$, ${\bar{p}}_{0}=0.5$, $\bar{\Delta }=3$, $\bar{k}=1$ and ${\bar{v}}_{0}=5$. Diverse contraction coefficients produce distinct limit cycles, as shown in Figure 5a, among which the critical contraction coefficient necessary for triggering the self-oscillatory pattern is identified as $C_{0cr}\approx 0.05$. When $C_{0}$ is below the critical value, the system is in a hovering pattern. The self-oscillatory pattern is triggered when $C_{0}>0.05$, e.g., $C_{0}=0.1$, $C_{0}=0.2$ and $C_{0}=0.3$. Figure 5b depicts that an elevation in the contraction coefficient tends to amplify the self-oscillation amplitude, whereas a reduction in frequency occurs; therefore, we can achieve the effect we want by adjusting $C_{0}$. As indicated by Equations (3), (14) and (19), increasing $C_{0}$ leads to a corresponding rise in the light-driven contraction, which subsequently improves buoyancy ${\bar{F}}_{H}$, ultimately raising A and f. As suggested by the outcomes, elevating $C_{0}$ can effectively improve the self-oscillatory effect.

### 4.3. Effect of Damping Coefficient

**Figure 6:**
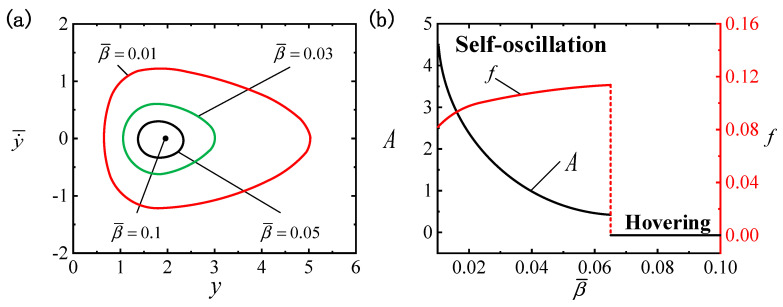
Effect of damping coefficient $\bar{\beta }$ on self-oscillation for $\bar{I}=0.4$, $C_{0}=0.3$, $\bar{g}=0.3$, ${\bar{p}}_{0}=0.5$, $\bar{\Delta }=3$, $\bar{k}=1$ and ${\bar{v}}_{0}=5$. (**a**) Limit cycles. (**b**) Effect of $\bar{\beta }$ on f and A. As $\bar{\beta }$ increases, A tends to decrease and f tends to increase.

Figure 6 describes how $\bar{\beta }$ influences the LCE ball system, where the remaining parameters are $\bar{I}=0.4$, $C_{0}=0.3$, $\bar{g}=0.3$, ${\bar{p}}_{0}=0.5$, $\bar{\Delta }=3$, $\bar{k}=1$ and ${\bar{v}}_{0}=5$. As plotted in Figure 6a, different damping coefficients lead to distinct limit cycles. ${\bar{\beta }}_{0cr}\approx 0.065$ is recognized as the critical damping coefficient that triggers the self-oscillatory pattern. When $\bar{\beta }$ surpasses the critical value, the damping dissipation is so excessive that the energy sourced from the external environment is inadequate to counteract it, resulting in a hovering pattern. The self-oscillatory pattern is triggered when ${\bar{\beta }}_{0}<0.065$, e.g., $\bar{\beta }=0.01$, $\bar{\beta }=0.03$ and $\bar{\beta }=0.05$. As reflected in Figure 6b, the growing damping coefficient $\bar{\beta }$ causes a drop in the self-oscillation amplitude and a boost in frequency, and it can be seen that adjusting $\bar{\beta }$ can bring the self-oscillation to the desired state. One can comprehend this phenomenon by examining the energy competition between the light energy input and damping dissipation. An increase in $\bar{\beta }$ contributes to heightened energy loss, which subsequently diminishes the amplitude. Consequently, to maximize energy harvesting in practical engineering, it is preferable to reduce the damping coefficient, thereby enhancing the absorption of light energy.

### 4.4. Effect of Gravitational Acceleration

**Figure 7:**
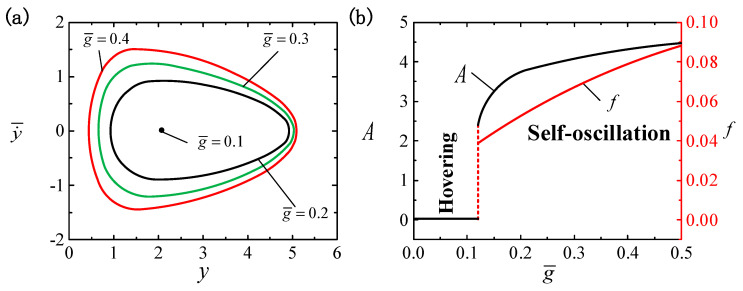
The effect of gravitational acceleration $\bar{g}$ on self-oscillation for $\bar{I}=0.4$, $C_{0}=0.3$, $\bar{\beta }=0.01$, ${\bar{p}}_{0}=0.5$, $\bar{\Delta }=3$, $\bar{k}=1$ and ${\bar{v}}_{0}=5$. (**a**) Limit cycles. (**b**) The effect of $\bar{g}$ on f and A. With the rise in $\bar{g}$, there is a noticeable upward trend in both A and f.

Figure 7 displays the effect of $\bar{g}$ on the LCE ball system, among which the other parameters are $\bar{I}=0.4$, $C_{0}=0.3$, $\bar{\beta }=0.01$, ${\bar{p}}_{0}=0.5$, $\bar{\Delta }=3$, $\bar{k}=1$ and ${\bar{v}}_{0}=5$. In Figure 7a, distinct limit cycles are plotted, with each corresponding to different values of gravitational acceleration. A critical gravitational acceleration of ${\bar{g}}_{cr}\approx 0.12$ is present to trigger the self-oscillatory pattern. When $\bar{g}$ is less than the critical value, the system is in a hovering pattern. The explanation for this finding lies in the energy balance between the energy input and damping dissipation, suggesting that the energy input cannot consistently make up for the energy lost due to damping dissipation, thereby preserving the self-oscillatory pattern. The self-oscillatory pattern is triggered when ${\bar{g}}_{0}>0.12$, e.g., $\bar{g}=0.51$, $\bar{g}=0.53$ and $\bar{g}=0.55$. As observed in Figure 7b, with an increasing $\bar{g}$ value, a noticeable growth in both the A and f of the self-oscillations is witnessed, so we can achieve the effect we want by adjusting $\bar{g}$. Hence, to optimize the conversion of light energy into mechanical energy, one could consider elevating the gravitational acceleration appropriately.

### 4.5. Effect of Air Density

**Figure 8:**
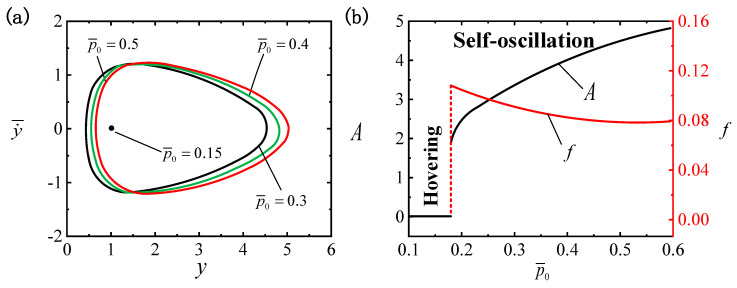
The effect of air density ${\bar{p}}_{0}$ on self-oscillation for $\bar{I}=0.4$, $C_{0}=0.3$, $\bar{\beta }=0.01$, $\bar{g}=0.3$, $\bar{\Delta }=3$, $\bar{k}=1$ and ${\bar{v}}_{0}=5$. (**a**) Limit cycles. (**b**) The effect of ${\bar{p}}_{0}$ on f and A. As ${\bar{p}}_{0}$ increases, A reflects a growth trend, whereas f experiences a decline.

Figure 8 presents the effect of ${\bar{p}}_{0}$ on the LCE ball system. In the numerical calculations, we set $\bar{I}=0.4$, $C_{0}=0.3$, $\bar{\beta }=0.01$, $\bar{g}=0.3$, $\bar{\Delta }=3$, $\bar{k}=1$ and ${\bar{v}}_{0}=5$. Different air densities result in distinct limit cycles, as shown in Figure 8a, among which the critical air density required to trigger the self-oscillatory pattern is identified as ${\bar{p}}_{0cr}\approx 0.18$. When ${\bar{p}}_{0}$ falls beneath the critical value, a hovering pattern occurs in the system. In contrast, the self-oscillatory pattern is triggered when ${\bar{p}}_{0}>0.18$, e.g., ${\bar{p}}_{0}=0.3$, ${\bar{p}}_{0}=0.4$ and ${\bar{p}}_{0}=0.5$. In Figure 8b, as the air density increases, the self-oscillation amplitude exhibits a tendency to increase, while the frequency gradually diminishes. That is to say, the smaller the air density ${\bar{p}}_{0}$, the more difficult it is to trigger self-oscillation. Correspondingly, the insights can be derived from Equations (14) and (19). An elevation in air density ${\bar{p}}_{0}$ contributes to a boost in buoyancy ${\bar{F}}_{H}$, which, in turn, amplifies the work performed by the buoyancy. Therefore, in practical applications, the efficiency of converting light energy into mechanical energy can be optimized by suitably raising the air density.

### 4.6. The Effect of the Bottom Illumination Zone Height

**Figure 9:**
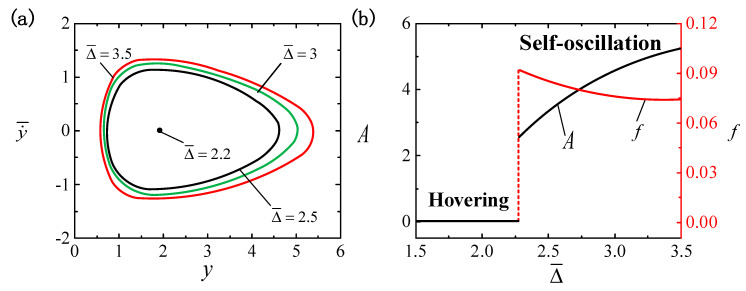
The effect of the bottom illumination zone height $\bar{\Delta }$ on self-oscillation for $\bar{I}=0.4$, $C_{0}=0.3$, $\bar{\beta }=0.01$, $\bar{g}=0.3$, ${\bar{p}}_{0}=0.5$, $\bar{k}=1$ and ${\bar{v}}_{0}=5$. (**a**) Limit cycles. (**b**) The effect of $\bar{\Delta }$ on f and A. An elevation in $\bar{\Delta }$ leads to an increase in A and a decrease in f.

Figure 9 depicts the effect of $\bar{\Delta }$ on the LCE ball system, where the other parameters are set as $\bar{I}=0.4$, $C_{0}=0.3$, $\bar{\beta }=0.01$, $\bar{g}=0.3$, ${\bar{p}}_{0}=0.5$, $\bar{k}=1$ and ${\bar{v}}_{0}=5$. Figure 9a shows that varying bottom illumination zone heights correspond to different limit cycles. There also exists a critical value of ${\bar{\Delta }}_{cr}=2.25$ to trigger self-oscillation. When $\bar{\Delta }\leq 2.25$, the system is in a hovering pattern. The self-oscillatory pattern is triggered when $\bar{\Delta }>2.25$, e.g., $\bar{\Delta }=2.5$, $\bar{\Delta }=3$ and $\bar{\Delta }=3$. As illustrated in Figure 9b, a rise in $\bar{\Delta }$ corresponds with an enhancement in A and a reduction in f. That is to say, the smaller the bottom illumination zone height $\bar{\Delta }$, the more difficult it is to trigger self-oscillation. Therefore, the improvement in self-oscillation requires the bottom illumination zone height $\bar{\Delta }$ to be maintained at an adequate level. The higher the bottom illumination zone, the longer the illumination duration, the more light energy is absorbed and the greater the self-oscillation amplitude. Hence, the bottom illumination zone height can be appropriately increased to promote the absorption of light energy.

### 4.7. Effect of Characteristic Coefficient

**Figure 10:**
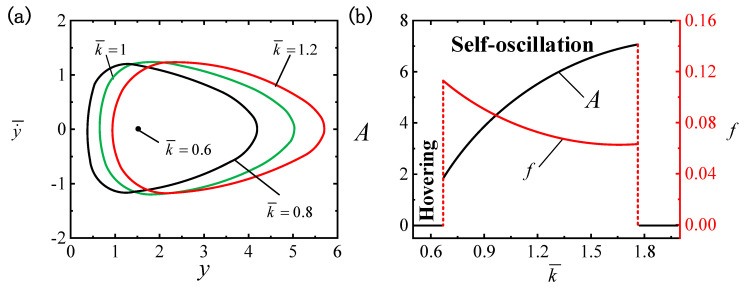
The effect of the characteristic coefficient $\bar{k}$ on self-oscillation for $\bar{I}=0.4$, $C_{0}=0.3$, $\bar{\beta }=0.01$, $\bar{g}=0.3$, ${\bar{p}}_{0}=0.5$, $\bar{\Delta }=3$ and ${\bar{v}}_{0}=5$. (**a**) Limit cycles. (**b**) The effect of $\bar{k}$ on f and A. With the increase in $\bar{k}$, there is a rise in A and a drop in f.

The effect of $\bar{k}$ on the LCE ball system is illustrated in Figure 10, where the remaining parameters are $\bar{I}=0.4$, $C_{0}=0.3$, $\bar{\beta }=0.01$, $\bar{g}=0.3$, ${\bar{p}}_{0}=0.5$, $\bar{\Delta }=3$ and ${\bar{v}}_{0}=5$. As displayed in Figure 10a, diverse characteristic coefficients produce distinct limit cycles. The two critical characteristic coefficients for triggering the transition between the hovering pattern and the self-oscillatory pattern are numerically determined as $\bar{k}\approx 0.67$ and $\bar{k}\approx 1.75$. When $\bar{k}\leq 0.67$ or $\bar{k}\geq 1.75$, the system is in a hovering pattern. The self-oscillatory pattern is triggered when $0.67<\bar{k}<1.75$, e.g., $\bar{k}=0.8$, $\bar{k}=1$ and $\bar{k}=1.2$. In Figure 10b, with the increase in $\bar{k}$, the A value of the self-oscillation reflects a growth trend, whereas f experiences a decline, but it should not be too large or too small; otherwise, it will affect the self-oscillating oscillation effect. Following Equation (19), an increase in the characteristic coefficient $\bar{k}$ leads to a rise in buoyancy, which subsequently leads to a growth in the work performed by buoyancy. Therefore, an appropriate increase in $\bar{k}$ is one of the effective measures to promote the absorption of light energy, but it should not be too large.

### 4.8. The Effect of Air Velocity at the Blower Outlet

**Figure 11:**
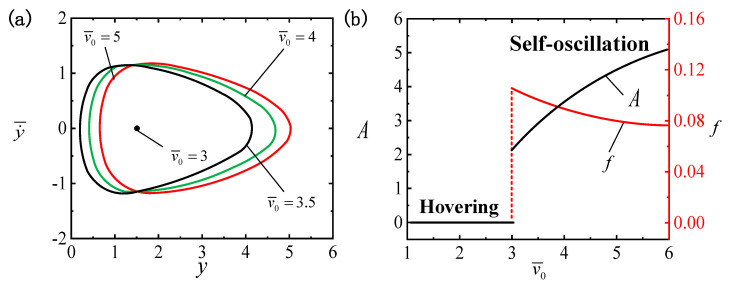
The effect of the vertical wind speed at the blower outlet ${\bar{v}}_{0}$ on self-oscillation for $\bar{I}=0.4$, $C_{0}=0.3$, $\bar{\beta }=0.01$, $\bar{g}=0.3$, ${\bar{p}}_{0}=0.5$, $\bar{\Delta }=3$ and $\bar{k}=1$. (**a**) Limit cycles. (**b**) The effect of ${\bar{v}}_{0}$ on f and A. As ${\bar{v}}_{0}$ increases, the A value of the self-oscillation increases, while f decreases.

Figure 11 depicts the effect of ${\bar{v}}_{0}$ on the LCE ball system. We assign the other parameters as $\bar{I}=0.4$, $C_{0}=0.3$, $\bar{\beta }=0.01$, $\bar{g}=0.3$, ${\bar{p}}_{0}=0.5$, $\bar{\Delta }=3$ and $\bar{k}=1$. Figure 11a illustrates that different vertical wind speeds at the blower outlet result in different limit cycles. A critical vertical wind speed of ${\bar{v}}_{0cr}\approx 3$ is present for triggering the transition between the hovering pattern and the self-oscillatory pattern. When ${\bar{v}}_{0}$ is below the critical value, the system enters the hovering pattern. The self-oscillatory pattern is triggered when ${\bar{v}}_{0}>3$, e.g., ${\bar{v}}_{0}=3.5$, ${\bar{v}}_{0}=4$ and ${\bar{v}}_{0}=5$. Evidently, the increase in ${\bar{v}}_{0}$ brings about elevated A and reduced f values for the self-oscillation, and ${\bar{v}}_{0}$ should not be too small; otherwise, it will be more difficult to trigger self-oscillation. As mentioned above, the buoyancy on the LCE ball is related to the vertical wind speed at the blower outlet and the air density. Therefore, increasing ${\bar{v}}_{0}$ will lead to a growth in the work performed by buoyancy. In practice, the conversion efficiency of light energy can be improved by an appropriate elevation in ${\bar{v}}_{0}$.

## 5. Conclusions

Self-oscillation enables continuous motion by transforming constant external stimuli into mechanical work, which lays the foundation for its wide application in engineering. To address the problems of a complex structure, difficult construction and control in the existing self-oscillatory systems, the current work creatively proposes a suspended LCE ball system consisting of a hollow LCE ball and an air blower, which can self-oscillate under stable illumination. Drawing from the theoretical model in conjunction with the dynamic LCE model, governing equations are derived, and then numerical calculations are carried out using the MATLAB software. As suggested by the numerical calculations, there involves two motion patterns, i.e., the hovering and the self-oscillatory patterns. Furthermore, we clarify the underlying mechanism for self-oscillation.

In addition, self-oscillation is affected by various system parameters. For instance, increasing light intensity $\bar{I}$, the contraction coefficient $C_{0}$, air density ${\bar{p}}_{0}$, the bottom illumination zone height $\bar{\Delta }$, the characteristic coefficient $\bar{k}$, and the vertical wind speed at the blower outlet ${\bar{v}}_{0}$ will typically lead to a rise in the self-oscillation amplitude and a drop in frequency. In contrast, with the increase in the damping coefficient $\bar{\beta }$, the amplitude experiences a decline, whereas the frequency shows an increase. An increase in gravitational acceleration $\bar{g}$ results in simultaneous growths in amplitude and frequency.

Inspired by contraction–expansion-induced self-oscillations, the Bernoulli principle and the Coanda effect, combined with the dynamic LCE model, the control equation of the system is established, and the system itself is established. This self-oscillating levitating LCE ball system may change the approach to energy harvesting, microsensors and aerial vehicles due to its structural simplicity, customizable dimensions, remote activation and active manipulation.

Regarding the current study, making an LCE ball was difficult due to the limitations of the experimental conditions, so it is hard to validate this theoretical exploration through experiments. This paper focuses on the theoretical aspects of self-excited oscillations in LCE balls. Considering that the results of this study are consistent with physical intuition, we feel that this LCE ball system can be experimentally verifiable in the future. To summarize, in the forthcoming work, the self-oscillatory behavior of the system will be validated through experimental approaches. We know that LCE materials exhibit distinct responses to different stimuli, e.g., for thermally driven self-excited bilinear pendulums [78], periodic oscillations can occur under temperature stimulation with a constant gradient. In addition, hydrogels and ionic gels also show different responses to light stimulation, such as light-responsive hydrogel-driven soft robots [79], so this system can be constructed by switching to other light-responsive materials. At the same time, we also believe that it is very important to pay attention to the LCE material itself, and more attention should be paid to this aspect in future theoretical research and experiments.

## Figures and Tables

**Table 1:** Material properties and geometric parameters.

Parameter	Definition	Value	Unit
I	Light intensity	0~100	kW/m^2^
m	Mass of hollow LCE ball	0.1–0.5	kg
β	Damping coefficient	0~1	kg/s
$C_{0}$	Contraction coefficient	0~0.4	/
$T_{0}$	Thermal relaxation time	0.01~0.5	s
g	Gravitational acceleration	0~10	m/s^2^
$p_{0}$	Air density	40~70	kg/m^3^
$r_{0}$	Initial radius of hollow LCE ball	0.05~0.25	m
$v_{0}$	Vertical wind speed at blower outlet	0~60	m/s
k	Characteristic coefficient	0~0.25	m
Δ	Height of bottom illumination zone	0.1~0.4	m
$\eta_{0}$	Light absorption constant	0.00022	m^2^/(s·W)

**Table 2:** Dimensionless parameters.

Parameter	$\bar{I}$	$C_{0}$	$\bar{\beta }$	$\bar{g}$	${\bar{p}}_{0}$	$\bar{k}$	$\bar{\Delta }$	${\bar{v}}_{0}$
**Value**	0~1	0~0.4	0~0.2	0~1	0.1~0.6	0.5~2.5	1~4	1~6

## Data Availability

The original contributions presented in the study are included in the article, further inquiries can be directed to the corresponding author.
